# Oral washes and tongue swabs for Xpert MTB/RIF Ultra-based tuberculosis diagnosis in people with and without the ability to make sputum

**DOI:** 10.21203/rs.3.rs-6225530/v1

**Published:** 2025-03-18

**Authors:** Loren Rockman, Shima Abdulgader, Stephanie Minnies, Zaida Palmer, Charissa C. Naidoo, Daphne Naidoo, Rouxjeane Venter, Gcobisa Ndlangalavu, Byron W.P. Reeve, Andrew M. Marino, Tim J. Bull, Alaina M. Olson, Rachel Wood, Gerard A. Cangelosi, Robin M. Warren, Grant Theron

**Affiliations:** Stellenbosch University; Stellenbosch University; Stellenbosch University; Stellenbosch University; Stellenbosch University; Stellenbosch University; Stellenbosch University; Stellenbosch University; Stellenbosch University; University of Pennsylvania; City and St. George’s University of London, London; University of Washington; University of Washington; University of Washington; Stellenbosch University; Stellenbosch University

**Keywords:** Xpert MTB/RIF Ultra, tuberculosis, oral washes, tongue swabs

## Abstract

**Background:**

Oral samples show promise for tuberculosis (TB) diagnosis. Data from different samples and people with sputum scarce TB are limited.

**Methods:**

We assessed Xpert MTB/RIF Ultra (Ultra) in symptomatic people at clinics (Cohort A, n=891) or at antiretroviral therapy (ART)-initiation without syndromic preselection (Cohort B, n=258). In Cohort A, we collected oral washes (OWs) and, separately, tongue swabs (flocked, foam with heat). In Cohort B, we collected OWs, three flocked tongue swabs (comparing one with heat to two pooled swabs) and, separately, buccal swabs, periodontal brushes. We offered sputum induction and did different culture methods on a subset of Cohort B tongue swabs.

**Results:**

In Cohort A, Ultra on OWs, flocked tongue and foam swabs had sensitivities of 80% (95% confidence interval 56, 94), 59% (53, 65) and 65% (58, 72) and high specificities. In Cohort B, OWs and single heated swabs had 71% (42, 92) and 64% (35, 87) sensitivity, respectively. Pooled tongue swabs, buccal swabs and periodontal brushes had low sensitivities. MGIT960 had the highest sensitivity [64% (35, 87)] of culture methods. Oral sampling detected TB in sputum-scarce people [Cohort A: 25% (7/28) flocked and foam swab-positive; Cohort B: 18% (10/56) OW-, 23% (13/56) single flocked swab-positive]. In Cohort B, this would at least double the people with a positive Ultra result (sputum or oral) if induction were unavailable.

**Conclusion:**

Ultra on OWs or foam tongue swabs has higher sensitivity than other oral-based approaches and detects sputum-scarce TB, resulted in more people diagnosed compared to Ultra on expectorated sputum.

## Background

Tuberculosis (TB), caused by *Mycobacterium tuberculosis* complex (*Mtb*), is a global pandemic (1). Molecular tests like Xpert MTB/RIF Ultra (Ultra; Cepheid, Sunnyvale, USA) and Truenat MTB Plus (Molbio Diagnostics, Goa, India) were designed for sputum. Non-sputum tests are urgently needed to reach the millions of people with TB undiagnosed each year (2–4).

Tongue swabs, which collect biofilm from the dorsum tongue, have data to support use with the Ultra and Truenat MTB Ultima (Ultima) tests: Ultra has sensitivities from 72–75% with high specificity, with similar performance described for Ultima (5). Furthermore, discrete choice studies show tongue swabs are more acceptable than sputum to some people despite potential sensitivity trade-offs (6). Importantly, a test with diminished sensitivity can increase diagnostic yield if the number of individuals tested is higher (7, 8), however, there are few empiric data in the context of oral samples (9).

Key gaps remain regarding the diagnostic potential of oral samples, including samples other than tongue swabs. Early proof-of-concept studies used in-house rather than commercially-available tests or, as tongue swabs are likely most beneficial in people unable to expectorate, paradoxically pre-selected people based on their ability to naturally produce sputum (10–13). Furthermore, the type of tongue swab (flocked, foam) and processing method may influence performance (14). Equally important, data from people with risk factors for TB not preselected based on symptoms, who are less able to make sputum and increasingly targeted as part of facility active case finding strategies, are scarce. More data are urgently needed to inform global policy.

Data are also scarce regarding *Mtb* culturability from tongue swabs. One study found 44–58% of swabs to be MGIT960 culture-positive in people with TB (15). Culturable *Mtb* from the swab could reinforce microbiological reference standards for diagnostic accuracy evaluations and provide material for drug susceptibility testing. Lastly, culture methods that do not involve harsh NALC-NaOH decontamination and are designed for paucibacillary samples may have added utility.

To address these knowledge gaps, we evaluated different types of oral samples, swabs, and processing methods for TB diagnosis in a HIV high burden setting where people were offered sputum induction. We hypothesized Ultra would detect *Mtb* in oral specimens with high concordance to sputum.

## Methods and Materials

### Ethics statement

This work was approved by the Health Research Ethics Committee of Stellenbosch University (N14/10/136, M20/06/017, M20/06/018) and City of Cape Town (10570). Written informed consent was obtained.

### Study Cohorts

#### Cohort A:

Adults (≥18 years) self-reporting with presumptive pulmonary TB symptoms meeting WHO criteria (at least one symptom for PLHIV, ≥2 for those without HIV) were recruited at clinics in Cape Town, South Africa.

#### Cohort B:

Antiretroviral therapy (ART) initiators (≥18 years), regardless of symptoms, were recruited in Cape Town as previously described (16, 17).

### Definitions

#### Cohort A:

People were classified as having TB using an extended microbiological reference standard (eMRS) if sputum was Ultra-positive or had *Mtb*-positive culture growth. Those categorized as not having TB had no positive sputum Ultra or culture results at least one negative result. Those missing a culture or Ultra result were, if other results negative, classified as not having TB.

#### Cohort B:

The eMRS consisted of two sputum cultures. If at least one culture was *Mtb*-positive, participants were classified as having TB. Participants with negative culture(s) did not have TB.

### Specimen collection

**Figure 1:**
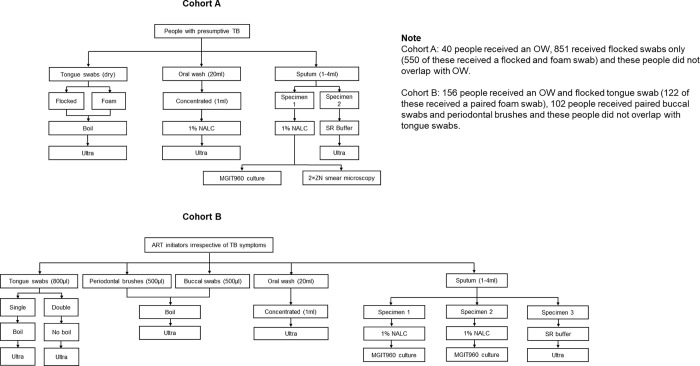
Flow chart showing enrolment, specimen collection and processing, and tests in Cohorts A and B.

All oral samples were collected prior to sputum collection after ≥30 min had passed since food or fluid was ingested and teeth brushed. Figure 1 summarises specimen collection and testing.

#### Oral washes

Each person (n=40 Cohort A, n=156 Cohort B) received two vials of 20 mL sterile water. One vial was used to rinse the mouth and discarded. The second vial’s contents were swirled in the mouth for a few seconds and spat into the vial. All samples were stored at −20 °C until processed. In Cohort A, OWs were collected from separate people than tongue swabs. In Cohort B, OWs were collected in everyone who gave tongue swabs and collection was done immediately after swab collection.

#### Tongue swabs

##### Cohort A:

One dry flocked swab (FLOQSwabs code 520C; Copan Italia S.p.A., Brescia, Italy) followed by a dry foam swab (Medline Industries, Northfield, USA) were consecutively collected from 851 and 550 people, respectively, by scraping the tongue dorsum for 10–15 s and placing the swab in a dry tube (550 people had both swab types). As a control for each person, an air swab was collected in the same space as the participant by waving a foam swab (flocked prior to the start of foam swab sampling) in the air for 10–15 s immediately before sampling.

##### Cohort B:

One flocked swab was collected in 800 μL Tris-EDTA (TE) buffer from 156 people. The last 122 were asked to provide an additional two flocked swabs subsequently pooled into a single tube with 800 μl TE buffer. A flocked air swab was done as for Cohort A. In both cohorts, samples were stored (−20 °C) until processed.

#### Buccal swabs and periodontal brushes

In Cohort B, paired buccal swabs and periodontal brushes (n=102) were collected (**Supplementary text pg. 2**) and stored (−20 °C) until processed. Collection occurred prior to the start tongue swab and OW collection.

#### Sputum

Cohort A participants were each asked to provide two sputa, while Cohort B participants were each asked to provide three sputa. Sputa were used for Ultra and MGIT960 culture [with 1% NALC-NaOH decontamination and MTBC confirmation using MTBDR*plus* (Bruker-Hain Diagnostics, Nehren, Germany); one culture in Cohort A, two in Cohort B]. Sputum induction was done (16, 18), however, it was only recorded whether people definitively required induction to make at least one ≥1 ml sputum in 584 Cohort A people and the 156 Cohort B people that gave tongue swabs. Figure 1 summarises specimens collected by Cohort.

### Specimen processing and testing

All processing was performed in a biosafety level 3 (BSL3) laboratory.

#### Oral wash testing using Ultra (Cohorts A and B)

In Cohort A, OWs were concentrated, decontaminated, and processed with sample reagent (SR; Cepheid) per **Supplementary text pg. 2**. Decontamination was not done in Cohort B.

#### Tongue swab testing using Ultra (Cohorts A and B)

##### Cohort A:

Swabs were removed from storage and placed into a heating block (100 °C, 10 min), after which TE buffer was added to 2 mL and the whole volume tested with Ultra (19) with no SR.

##### Cohort B:

Single flocked swabs in TE buffer were removed from storage and immediately boiled (100°C, 10 min) followed by SR addition (1.6 mL to 800 μL sample) and Ultra (19). For the 122 people who also gave a double swab, no heating was done, 2:1 SR added, and Ultra done **(Supplementary Figure 1).**

###### Air swab controls in people who had tongue swabs (Cohorts A and B)

Air swabs was processed and tested with Ultra using the same procedure as tongue swabs every tenth patient. If a participant had a positive tongue swab their air swab was tested.

###### Buccal swabs and periodontal brushes (Cohort B)

Stored samples were removed, processed, and tested with Ultra using the same procedure as tongue swabs in Cohort B.

#### Tongue swab culture (Cohort B)

We did different types of culture (MGIT960, TiKa (20), early bactericidal activity (EBA) (21); methodology in **Supplementary text pg. 2**) on single flocked tongue swabs (without heat inactivation). Speciation on positive growth was done using Ultra on a concentrated MGIT960 tube (22).

### Analysis

Methods and reporting are per STARD guidelines (23). Diagnostic accuracy metrics were calculated using Excel (Microsoft, Redmond, USA) and compared using prtest (24) in STATA version 16.0 (StataCorp, Texas, USA). The effect on sensitivity and specificity of Ultra trace results removed or reclassified to Ultra-negative in 2×2 tables was evaluated. Continuous data were compared with GraphPad Prism version 8.0.1 (GraphPad Software, San Diego, USA), also used for linear regression and correlation analysis. Diagnostic yield (DYT, diagnostic yield in those tested; DYD, diagnostic yield in those diagnosed) was calculated as described (8) and defined in **Supplementary text pg. 3.** Morbidity score information (TBscoreII) was collected (25). Venn diagrams were made using Interactivenn (26). In Cohort B, people were designated asymptomatic based on the WHO four symptom screen (27). P-values ≤0.05 were significant. Unsuccessful results are those not positive or negative by any test. We compared SPC C_T_ values from Ultra to measure inhibition (lower SPC C_T_s mean less inhibition) (28).

## Results

### Participant demographics

People in Cohort A were, compared to Cohort B, more likely to be female, have higher morbidity, and more likely to have previous TB (Table 1). In both cohorts, people with TB were more likely to be male and have higher morbidity.

#### Diagnostic accuracy of Ultra

**Figure 2:**
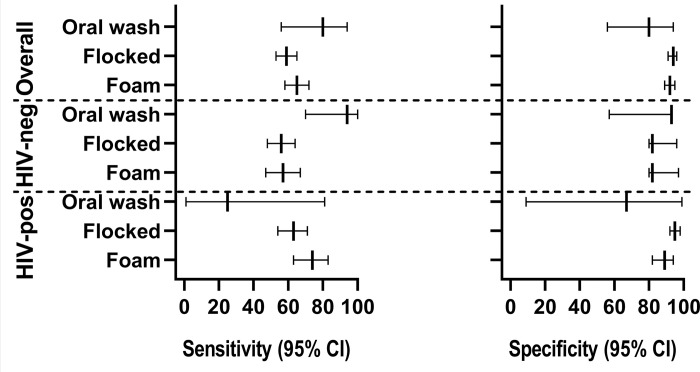
Forest plot of Ultra sensitivity and specificity on different oral samples in Cohort A, overall and stratified by HIV. Oral washes, foam swabs, and flocked swabs has the highest sensitivity.

**Figure 3:**
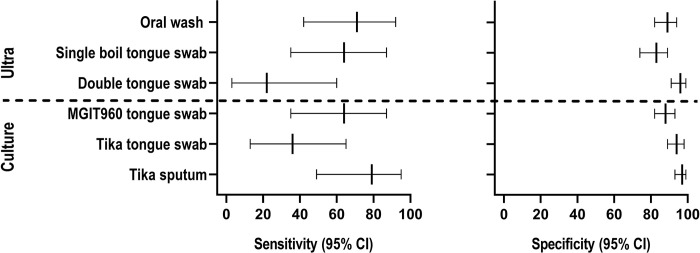
Forest plot of Ultra and culture sensitivity and specificity on oral samples in Cohort B. OWs had the highest sensitivity of oral samples.

Data are summarised in Figures 2 and 3. All air swabs were negative.

##### Cohort A

OWs: No unsuccessful results occurred. Sensitivity was 80% (56, 94) and specificity 80% (56–94). Sensitivity was higher among those without HIV compared to PLHIV [94% (70–100) vs 25% (1–81); p=0.002] (Table 2). Four false-positive results occurred (all trace semi-quantitation, two previous TB).

Tongue swabs: 3% (25/851) of flocked swabs and 2% (13/550; p=0.611) of foam swabs had unsuccessful results (3 both, 22 flocked only, 10 foam only; mostly overpressure errors). Flocked swabs had lower sensitivity than foam swabs [59% (53–65) vs. 65% (58–72); p=0.001] with high specificities [94% (91–96) vs. 92% (89–95); p=0.100] (Table 2). Amongst false-positive swabs, 26% (9/34) of flocked swabs and 39% (11/28) of foam swabs were from people programmatically empirically treated (no positive bacteriology at treatment start). Different trace recategorization strategies resulted in small sensitivity decreases and large specificity increases (**Supplementary Table 1**).

##### Cohort B

OWs: No unsuccessful results occurred. Sensitivity was 71% (42–92) and specificity 92% (86–96). Of the 12 false positives, all were Ultra semi-quantitation category trace. 17% (2/12) were Ultra flocked tongue swab positive.

Buccal swabs and periodontal brushes: No unsuccessful results occurred. Sensitivity was 7% (0–34) and 14% (2–43), respectively, with 98% (92–100) specificity for both (**Supplementary Table 2**).

Tongue swabs: 1% (2/156) of single swabs, and 1% (1/122; p=0.711) double swabs generated unsuccessful results. In head-to-head analyses, single vs. double swab sensitivity was 67% (30–93) and 22% (3–60; p=0.068) and specificity 82% (74–89) vs. 96% (91–99; p=0.009) (Table 3)). Alternative evidence of TB occurred in 23% (6/26) of false-positive single flocked swabs [two sputum Ultra-positive, one Alere Determine TB LAM Ag (Abbott, USA)-positive, three positive MGIT960 tongue swab culture] and 25% (1/4) of false-positive double swabs (sputum Ultra-positive). Different trace recategorization strategies resulted in small sensitivity decreases and large specificity increases (**Supplementary Table 3** ).

Asymptomatic TB: 58% (150/258) of people were asymptomatic and, of these, 6% (9/150) had TB. 33% (3/9), 22% (2/9) and 0% (0/9) were positive using OW, single flocked swab and double flocked swabs, respectively.

#### Diagnostic accuracy of tongue swab culture in Cohort B

MGIT960 and TiKa had 64% (35–87) vs. 36% (13–65; p=0.131) sensitivity, while specificity was 88% (82–93) vs. 94% (89–98; p=0.060) (Supplementary **Table 4**). For comparison, sputum TiKa culture sensitivity and specificity were 79% (49–95; p=0.022 vs. TiKa on tongue swabs) and 97% (93–99; p=0.238), respectively. In the subset who underwent tongue swab EBA culture, sensitivity and specificity were 33% (4–78) and 95% (85–99).

### Yield of Ultra

**Figure 4:**
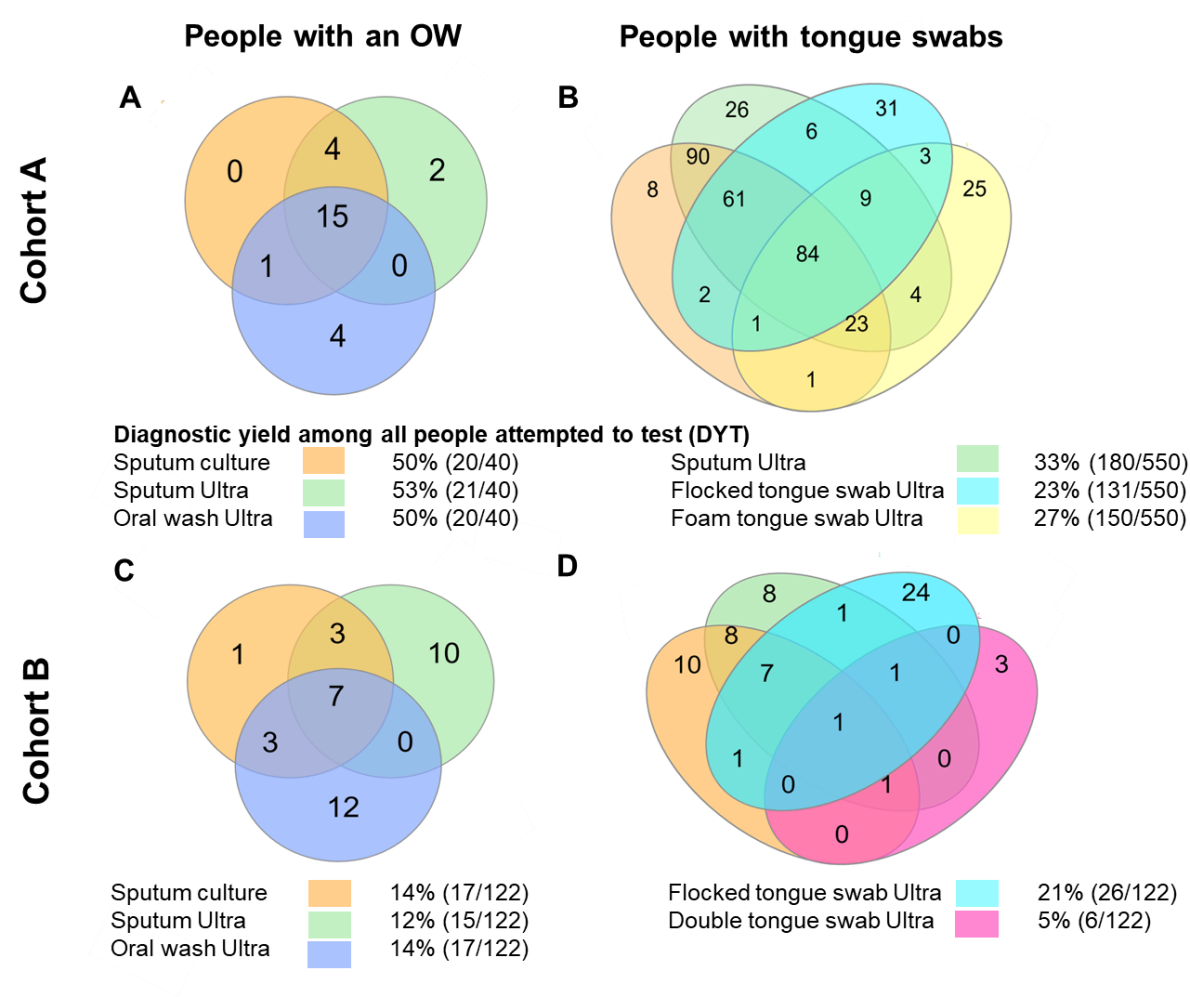
Number of people with a positive result in Cohorts A and B. In both cohorts, more people were detected exclusively by OW Ultra than any other test. Each oral sampling method also detected people missed by other approaches. The first column includes people with an OW, whereas the second includes people with tongue swabs (in Cohort B, OWs and tongue swabs were in the same people but are disaggregated here for clarity).

Figure 4 shows people who tested positive by Ultra on different samples, as well as sputum culture. Ultra yield metrics are compared in **Supplementary Table 5** Similar patterns for DYD occurred as those described for DYT. We also calculate yields from tongue swab culture, **(Supplementary text pg. 4),** which were low.

#### Cohort A

Overall: OW DYT (50%, 20/50) was like that for sputum. Foam tongue swabs had a higher DYT (27%, 150/550) point estimate than flocked tongue swabs (23%, 131/150) but this did not reach significance. Sputum DYT was significantly higher [33% (180/550] than flocked and foam swabs.

If sputum induction were unavailable: No one with an OW had sputum induction information. In people with both tongue swab types, 5% (28/550) could not expectorate sputum. 25% (7/28) and 25% (7/28) were foam or flocked tongue swab positive. If tongue swabs were done in people who could not expectorate sputum, people bacteriologically diagnosed rapidly would increase from 175 (Ultra-positive on expectorated sputum) to 182 for flocked and foam swabs, [4% (2–6) increase].

#### Cohort B

Overall: Amongst people with all three sample types, DYT point estimates were highest for single flocked tongue swab compared to OWs and double tongue swabs [21% (26/122), 14% (17/122), 5% (6/122)]. Sputum DYT was 12% (5/122).

If sputum induction were unavailable: In people who had all three sample types (OWs, single and double swabs), 35% (43/122) could not expectorate sputum, of which 16% (7/43), 26% (11/43) and 5% (2/43) were positive on each oral sample type. If oral sampling was done in people who could not expectorate sputum, bacteriological diagnoses would change from 8 (Ultra-positive on expectorated sputum) to 15, 19, and 10 for OWs, single swabs, and double swabs, respectively [+88% (82–93), +138% (130–145) and 25% (17–33) respectively. If induction were unavailable, sputum DYT decreased to 7% (8/122), lower than OWs [14% 17/122; p=0.057] and single flocked swabs [21% (26/122); p=0.0009].

##### Ultra inhibition

In Cohort A, oral washes had less PCR inhibition than sputum, however, this did not occur in Cohort B. No other differences of note occurred across sample types **(Supplementary text pg. 4).**

## Discussion

Our key findings are: 1) OW Ultra had the highest sensitivity amongst the methods tested (71–80%), 2) Ultra on foam swabs had higher sensitivity than flocked swabs (65% vs. 59%; Cohort A), 3) Ultra on oral samples swabs diagnosed TB in many people who could not naturally expectorate, permitting yield to exceed that of sputum-based testing in Cohort B where sputum scarcity was more common, and 4) other approaches (Ultra on double tongue swabs, buccal swabs, and periodontal brushes) were suboptimal. These data suggest oral sampling, especially oral washes and foam swabs, can improve the diagnosis of TB, especially when sputum scarcity is accounted for.

OW Ultra had a higher sensitivity point estimate than other approaches. To our knowledge, we report the first study using OW Ultra for TB diagnosis. Previous studies (29, 30) used in-house PCR methods applied to OW reported sensitivities of 77% and 88%, broadly our findings. We suggest OWs are included as a comparator in research on tongue swabs for TB diagnosis going forward as, unlike swabs, they require less processing.

We observed a small sensitivity increase with foam swabs compared to flocked swabs, likely attributable to the larger amount of biomass bound to the foam swab (15, 31). Foam swabs have an added advantage in that they are relatively cheap (0.14 USD per swab) compared to flocked swabs. On a practical note, however, participants report foam swabs make their tongues feel “dry”. Furthermore, the lack of a breakpoint means swab heads need to be cut off, however, this is addressable. The magnitude of the sensitivity improvement from foam swabs may, vs. flocked swabs, be even greater in people with earlier stage disease.

We showed Ultra on swabs can detect TB in people who cannot make. As TB testing programmatically expands, including to people with early stage potentially subclinical TB, the proportion of people with sputum scarce TB that require testing will increase. Thus, our study addresses a key gap: most studies on oral samples for TB diagnosis have either not recruited people with sputum scarce TB or have offered induction and not been able to disaggregate performance in people who, without induction, cannot expectorate. This is important because, even if tongue swabs perform well in sputum expectorators, it is hard to justify not testing sputum if it is available. Our data are thus novel in that they give performance data in a type of person most likely to benefit from non-sputum tests. For example, in Cohort B, the number of people with a positive Ultra result would at least double with the use of oral sampling in sputum scarce.

We compared a single flocked swab with heat lysis, which had high sensitivity in earlier work using contrived samples (32), to a double flocked swab without heat lysis, which had lower sensitivity. This suggests heating is critical to release DNA. It remains to be seen if heating with a double swab improves sensitivity further, however, given our results from foam swabs, it is likely that steps that input material would be beneficial, providing heat lysis occurs. In addition to the double swab method, we evaluated other specimen types (buccal swabs, periodontal brushes), as well as different tongue swabs culture methods, however, neither were promising.

Our study has strengths and limitations. Recruitment was programmatic in nature in that it included people with symptoms and non-symptom-based risk factors for which South African guidelines (33, 34) require molecular testing, even if they were not yet symptomatic and/or could not expectorate sputum. More data are needed in these groups where oral sample testing is likely to be most impactful (7). Different processing methods, such as those which maximise time to recover DNA buccal swabs and periodontal brushes were in buffer for short periods) should be further explored alongside OWs, which notably does not require heating. Lastly, the inclusion of people who cannot make sputum naturally and crucially the availability of induction information are strengths.

In conclusion, Ultra on oral samples – especially foam tongue swabs and OW – is sensitive and highly specific and can significantly increase the overall number of people with a rapid positive bacteriological result when applied to people who cannot naturally make sputum.

## Figures and Tables

**Table 1:** Demographic and clinical characteristics. Data are n/N (%) or median (IQR).

	Cohort A			Cohort B		
	Overall (n=891)	Definite TB (n=315)	Non-TB (n=576)	Overall (n=258)	Definite TB (n=28)	Non-TB (n=230)
**Demographic**						
Age (years)	38 (30–48)	38 (30–47)	38 (30 – 48) p=0.364*	37 (31–44) p=0.956^€^	36 (31–44) p=0.378^€^	39 (31 –43) p=0.352* p=0.002^€^
Female	445/891 (50)	131/315 (42)	314/576 (55) p=0.002*	156/258 (60) **p=0.003**^€^	11/28 (39) p=0.813^€^	145/230 (63) p=0.015* p=0.027^€^
**Clinical**						
HIV-positive	355/891 (40)	136/315 (44)	219/576 (38) p=0.133*	258/258 (100) **p<0.0001**^€^	28/28 (100) **p<0.0001**^€^	230/230 (100) **p<0.0001**^€^
CD4 count (cells/μL)	305 (269–903)	582 (250–945)	305 (290–605) p=0.587*	293 (143–434) **p<0.0001**^€^	59 (22–135) **p<0.0001**^€^	299 (171 – 438) p=0.013* p<0.0001^€^
TBScoreII	3 (2–3)	3 (3–4)	3 (2–3) p<0.0001*	2 (0–2) **p<0.0001**^€^	3 (2–4) **p=0.001**^€^	1 (0–2) p=0.0006* p<0.0001^€^
Previous TB	398/891 (45)	114/315 (36)	284/576 (49) p=0.0002*	26/258 (10) **p<0.0001**^€^	2/28 (7) **p=0.002**^€^	24/230 (10) p=0.585* p<0.0001^€^

Within column p-values:

* Definite TB vs. Non-TB

€ Across cohorts for people of the same TB status

Abbreviation: CD4, cluster of differentiation 4; HIV, human immunodeficiency virus; IQR, interquartile range

**Table 2:** Diagnostic accuracy of Ultra on OW or tongue swabs compared to an eMRS for the detection of TB stratified by HIV status in Cohort A. Data are %, (95% CI), and n/N.

	Overall^β^ (n=891)				HIV-negative (n=526)				HIV-positive (n=355)		
	Sensitivity	Specificity	PPV	NPV	Sensitivity	Specificity	PPV	NPV	Sensitivity	Specificity	PPV
**Oral wash**	80 (56, 94) 16/20	80 (56, 94) 16/20	80 (56, 94) 16/20	80 (56, 94) 16/20	94 (70, 100) 15/16	93 (57, 96) 14/17	83 (59, 96) 15/18	93 (68, 100) 14/15	25 (1, 81) 1/4 p=0.002*	67 (9, 99) 2/3 p=0.531*	50 (1, 99) 1/2 p=0.264*
**Flocked swab**	59 (53, 65) 164/277	94 (91, 96) 515/549	83 (77, 88) 164/198	82 (79, 85) 515/628	56 (48, 64) 84/150	82 (90, 96) 307/329	79 (70, 87) 84/106	82 (78, 86) 307/373	63 (54, 71) 78/124 p=0.420*	95 (92, 98) 204/214 p=0.660*	89 (80, 94) 78/88 p=0.416*
**Foam swab**	65 (58, 72) 123/188 **p=0.039**^€^	92 (89, 95) 321/349 p=0.100^€^	81 (74, 87) 123/151 p=0.679^€^	83 (79, 87) 321/386 **p=0.007**^€^	57 (47, 67) 58/101 p=0.385^€^	82 (90, 97) 197/209 p=0.100^€^	83 (72, 91) 58/70 p=0.617^€^	82 (77, 87) 197/240 p=0.117^€^	74 (63, 83) 62/84 p=0.164* p=0.013^€^	89 (82, 94) 119/134 p=0.660* p=0.100^€^	81 (70, 89) 62/77 p=0.314* p=0.906^€^

Within row p-values:

* HIV-negative vs. HIV-positive

Within column p-values:

€ vs. flocked swab

Abbreviations: CI, confidence interval; eMRS, extended microbiological reference standard; HIV, human immunodeficiency virus; NPV, negative predictive value; OW, oral wash; PPV, positive predictive value; Ultra, Xpert MTB/RIF Ultra

β Ten people had unknown HIV status

**Table 3:** Diagnostic accuracy of Ultra on OW or tongue swabs compared to a double sputum culture as an eMRS for TB detection in Cohort B. Data are %, 95% CI, and n/N.

	Head-to-head (n=122)				Non-head-to-head (n=156)			
	Sensitivity	Specificity	PPV	NPV	Sensitivity	Specificity	PPV	NPV
**Oral wash**	56 (21, 86) 5/9	89 (82, 94) 101/113	29 (10, 56) 5/17	96 (91, 99) 101/105	71 (42, 92) 10/14 p=0.435*	92 (86, 96) 130/142 p=0.556*	45 (24, 68) 10/22 p=0.307*	97 (93, 99) 130/134 p=0.725*
**Single boiled tongue swab**	67 (30, 93) 6/9 p=0.065^Ω^	82 (74, 89) 91/110 p=0.151^Ω^	23 (9, 44) 6/26 p=0.695^Ω^	97 (91, 99) 91/94 p=0.864^Ω^	64 (35, 87) 9/14 p=0.907* p=0.705^Ω^	81 (74, 87) 114/140 p=0.79* p=0.018^Ω^	26 (12, 43) 9/35 p=0.813* p=0.143^Ω^	96 (90, 99) 114/119 p=0.700* p=0.609^Ω^
**Double tongue swab**	22 (3, 60) 2/9 p=0.160^Ω^ p=0.068^≠^	96 (91, 99) 108/112 **p=0.042**^Ω^ **p=0.009**^≠^	33 (4, 78) 2/6 p=0.858^Ω^ p=0.639^≠^	94 (88, 98) 108/115 p=0.480^Ω^ p=0.395^≠^	22 (3, 60) 2/9 **p=0.022**^Ω^ **p=0.048**^≠^	96 (91, 99) 108/112 p=0.117^Ω^ **p=0.0004**^≠^	33 (4, 78) 2/6 p=0.595^Ω^ p=0.729^≠^	94 (88, 98) 108/115 p=0.247^Ω^ p=0.526^≠^

Within row p-values:

* Head-to-head vs. Non-head-to-head

Within column p-values:

Ω vs. OW

≠ vs. single boiled swab

Abbreviations: CI, confidence interval; eMRS, extended microbiological reference standard; NPV, negative predictive value; OW, oral wash; PPV, positive predictive value; Ultra, Xpert MTB/RIF Ultra
